# A Clinical Case

**Published:** 1890-09

**Authors:** W. A. Yingling

**Affiliations:** Nonchalanta, Kansas


### A CLINICAL CASE.

W. A. YINGLING, M. D., NONCHALANTA, KANSAS.

A. J. M., æt. thirty-eight, on February 2d, 1890, at about five P. M. threw a plank to one side, which, striking against a wire fence, bounded back with such force as to drive a large, rusty spike through the left foot, near the arch of the instep, but without passing through the bone, as it glanced to the inside of the foot. His son came to me at eight P. M., same evening, giving symptoms as follows: A few moments after the accident the patient felt stiffening pains in the foot, running up the leg, rapidly increasing in severity. Great chilliness, with chattering of the teeth soon followed. Lower jaw became somewhat stiff; general shivering; neck felt stiff; great anxiety; "can't endure it much longer." I sent some pellets of *Ledum*$^{3x}$, to be taken internally, and a dram vial of Calendula$^{3x}$, one-fourth to be put in a tumbler of water and a cloth wet with this kept on the foot. The report the next day was rapid improvement from the first dose. In thirty-six hours he was walking about, yet the foot felt somewhat tender. Entire and rapid recovery without bad results.

What would the results have been without Ledum for the punctured wound? Does effect follow the cause? If the threatened tetanus was the result of the rusty spike, why is not the cure the effect of the homœopathic remedy? Does Homœopathy cure? If not, then effect is not the result of cause, the effect would have taken place "any way."

